# Expression and Activity of a Novel Cathelicidin from Domestic Cats

**DOI:** 10.1371/journal.pone.0018756

**Published:** 2011-04-12

**Authors:** Brian C. Leonard, Hiutung Chu, Jennifer L. Johns, Richard L. Gallo, Peter F. Moore, Stanley L. Marks, Charles L. Bevins

**Affiliations:** 1 Department of Microbiology and Immunology, UC Davis School of Medicine, Davis, California, United States of America; 2 Department of Pathology, Microbiology and Immunology, UC Davis School of Veterinary Medicine, Davis, California, United States of America; 3 Division of Dermatology, University of California San Diego and VA San Diego Healthcare System, San Diego, California, United States of America; 4 Department of Medicine and Epidemiology, UC Davis School of Veterinary Medicine, Davis, California, United States of America; University of South Florida College of Medicine, United States of America

## Abstract

Cathelicidins are small cationic antimicrobial peptides found in many species including primates, mammals, marsupials, birds and even more primitive vertebrates, such as the hagfish. Some animals encode multiple cathelicidins in their genome, whereas others have only one. This report identifies and characterizes feline cathelicidin (feCath) as the sole cathelicidin in domestic cats (*Felis catus*). Expression of feCath is predominantly found in the bone marrow, with lower levels of expression in the gastrointestinal tract and skin. By immunocytochemistry, feCath localizes to the cytoplasm of neutrophils in feline peripheral blood. Structurally, the mature feCath sequence is most similar to a subgroup of cathelicidins that form linear α-helices. feCath possesses antimicrobial activity against *E. coli* D31, *Salmonella enterica* serovar Typhimurium (IR715), *Listeria monocytogenes* and *Staphylococcus pseudintermedius* (clinical isolate) similar to that of the human ortholog, LL-37. In contrast, feCath lacks the DNA binding activity seen with LL-37. Given its similarity in sequence, structure, tissue expression, and antimicrobial activity, the cathelicidin encoded by cats, feCath, belongs to the subgroup of linear cathelicidins found not only in humans, but also non-human primates, dogs, mice, and rats.

## Introduction

Antimicrobial peptides (AMPs) are small, cationic molecules synthesized by epithelial cells and leukocytes throughout the animal kingdom [1]. Depending on the specific molecule in question, these peptides are either expressed constitutively or upregulated when exposed to inflammatory stimuli or pathogens. These key effector molecules of innate immunity typically possess antimicrobial activity against bacteria, viruses, fungi and/or parasites [1], [2]. In addition to their direct killing capacity, AMPs can participate in other immune functions such as chemotaxis, wound repair, inhibition of apoptosis and modulation of TLR ligand signaling [1], [2]. The two major families AMPs in mammals are defensins and cathelicidins.

Cathelicidins are a unique group of antimicrobial peptides with respect to their amino acid sequence and structure. The precursor prepropeptide of this family of antimicrobials contains a signal sequence region, a propeptide domain and a mature peptide at the C-terminus [3]. The propeptide is similar to, and named after, a protease inhibitor, cathelin. Hence, cathelin (or cathepsin-L inhibitor) is the unifying feature of the cathelicidins and the amino acid sequence of this prodomain is highly conserved across species [4], [5]. In striking contrast, the C-terminal region, coding for the mature peptide, is often highly divergent from species to species. The mature peptides can be classified into four major subgroups: α-helical peptides, proline and arginine-rich peptides, tryptophan-rich peptides, and lastly, peptides with disulfide bonds [3]. In addition to differences in mature peptide sequences, there are striking differences in the number of cathelicidins encoded by the genomes of various species. Humans, mice, rats and dogs encode a single cathelicidin, whereas other species, such as cows, sheep and pigs, express multiple cathelicidins [6]. In those species with multiple cathelicidins, multiple genes, rather than alternative splicing of primary transcripts, accounts for the diversity. In species where only one cathelicidin is encoded, the mature peptide falls into the α-helical subgroup, however, in other animals, cathelicidins have diverse structure of the mature peptide.

Human cathelicidin is variously named hCAP18 (full length peptide of 18 kDa) or LL-37 (mature peptide of 37 residues beginning with two leucine residues). hCAP18/LL-37 mRNA is expressed in the bone marrow [7] and the peptide localizes to the specific granules of neutrophils [8]. Expression is also found in monocytic cells and lymphocytes [9]. In addition, the hCAP18/LL-37 expression is found in nonhematopoietic cells such as epidermis [10], as well as other epithelial cells such as the gastrointestinal tract [11]. Expression of hCAP18/LL-37 is constitutively expressed to high levels in the human bone marrow, and at much lower levels in the skin. LL-37 has been shown to inhibit the growth of both Gram-positive (ex. *Staphylococcus aureus* and *Listeria monocytogenes*) and Gram-negative bacteria (ex. *Salmonella enterica* serovar Typhimurium and *Escherichia coli*) at micromolar concentrations [12]. Interestingly, vitamin D_3_ can induce hCAP18/LL-37 expression in keratinocytes and monocytes/macrophages, whereas butyrate can induce its expression in colonocytes [13], [14].

In addition to antimicrobial activity, many cathelicidins often have varied activities including promotion of wound healing [12], sequestering LPS [12], and stimulating chemotaxis [15]. Moreover, links have been established between LL-37 and inflammatory disorders, including psoriasis and systemic lupus erythematosus [10], [16], [17]. For example, LL-37 can bind to extracellular host DNA from damaged or dead cells, resulting in a complex that can be recognized by TLR9 in the endosomal compartment of plasmacytoid dendritic cells (pDCs) [16]. Activation of TLR9 in pDCs causes upregulation and release of type I interferon into the local environment, and can potentially exacerbate existing inflammation.

To further our understanding of innate effector molecules, we sought to identify and characterize cathelicidins in the domestic cat. Our data will show that the cat encodes a single cathelicidin, termed feCath, which has high sequence similarity to cathelicidins found in dogs, mice, rats and humans. We examined the expression of feCath mRNA and peptide. Antimicrobial and DNA binding activity was also evaluated. Our findings support the conclusion that feCath fits into a subgroup of linear α-helical cathelicidins with dogs, mice, rats and humans, based on sequence and structural similarities, sites of expression, and antimicrobial activity.

## Materials and Methods

### Tissue

Feline tissue was procured from client-owned cats, euthanized at UC Davis School of Veterinary Medicine for reasons unrelated to our study, which were processed for necropsy and labeled as unrestricted for use in research. Intestinal tissue (duodenum, proximal and distal jejunum, ileum, colon), skin and bone marrow were obtained. Tissues were immediately placed into RNAlater (Ambion, Applied Biosystems, Foster City, CA) and rocked at room temperature for 24 hours. Preserved specimens were then frozen at −80°C, submerged in this reagent, until further processing. Peripheral blood was obtained from excess blood from donor cats at the UC Davis School of Veterinary Medicine. Blood was directly collected into EDTA containing vacutainer tubes, refrigerated and used the following day.

### RNA Isolation and cDNA Synthesis

Tissue samples preserved in RNAlater were thawed and then homogenized in a guanidine thiocyanate buffer. Total RNA was isolated by cesium chloride centrifugation as described by Wehkamp *et al* [18]. Isolated total RNA was quantified by UV quantification at 260 nm using a spectrophotometer (Nanodrop ND-1000, Thermo Scientific, Wilmington, DE) and 5.0 µg of total RNA was reverse transcribed into cDNA using oligo-(dT) according to manufacturers protocol (Superscript II, Invitrogen, Carlsbad, CA). Subsequent to RNase-H treatment, cDNA was purified using column adsorption chromatography (PCR Purification Kit, Qiagen, Valencia, CA). Eluant cDNA was diluted to an equivalent of 10 ng/µl in 10 mM Tris-HCl based on the initial input concentration of total RNA.

### RACE Amplification

Isolated RNA from feline bone marrow was reverse transcribed into cDNA according to manufacturers protocol (Superscript II, Invitrogen) using oligo d(T) linked to an adapter sequence (Table 1: 3′-RACE Adapter Primer). To identify cathelicidins expressed in feline bone marrow, a 3′-RACE strategy was employed using three approaches. The first was to amplify cDNA using PCR primers (Table 1: Tossi 2A, Tossi 2B, Tossi 2C, Tossi 4A) described by Tossi *et al* [19], the second approach was to amplify cDNA with PCR primers used by Gallo and colleagues (Table 1: Gallo 1, Gallo 2)[20], and the last approach used a specific PCR primer (Table 1: K9CATH Sig Seq) that correspond to nucleotides encoding the signal sequence of canine cathelicidin (K9CATH, NM_001003359.1). Primer AP1 (Table 1: Primer AP1) was used as the antisense primer for all three approaches. PCR products were resolved by agarose gel electrophoresis (1.5% w/v) and bands were detected by ethidium bromide staining. To determine the 5′ sequence of the feCath transcript, cDNA was generated from bone marrow total RNA using 5′-RLM RACE kit (Ambion), ligating an RNA anchor sequence (Table 1: 5′-RACE Adapter Primer) to the 5′ end of the full-length transcripts. The RNA was then reverse transcribed using oligo-d(T) as above, and cDNA was amplified with an antisense gene-specific primer (Table 1: feCath 5′-RACE), based on the feCath sequence, and a 5′-RACE Outer Primer (Table 1: 5′-RACE Outer Primer). PCR products were column purified (Qiagen), and sequenced directly or ligated into pBluescript, subcloned into DH5α cells and sequenced.

**Table 1:** Oligonucleotide Primer Sequences.

Oligonucleotide Primer Name	Sequence
3′-RACE Adapter Primer	5′-CCATCCTAATACGACTCACTATAGGGCTCGAGCGGC(T)17-3′
Tossi 2A	5′-GCGAATTCTGTGAGCTTCAGGGTG-3′
Tossi 2B	5′-CCGAATTCAGCTACAGGGAGGCCGT-3′
Tossi 2C	5′-CCGAATTCAGTGTGACTTCAAGGA-3′
Tossi 4A	5′-AAGAATTCGGAGACTGGGACCATG-3′
Gallo 1	5′-TCGGAAGCTAATCTCTAC-3′
Gallo 2	5′-CTGGACCAGCCGCCCAAG-3′
K9CATH Sig Seq	5′-GGTGGTCACTGTTGCTACTGCT-3′
Primer AP 1	5′-CCATCCTAATACGACTCACTATAGGGC-3′
5′-RACE Adapter Primer	5′-GCUGAUGGCGAUGAAUGAACACUGCGUUUGCUGGCUUUGAUGAAA-3′
feCath 5′-RACE	5′-GATGTCAAAGTAGCCCCTGTTCC-3′
5′-RACE Outer Primer	5′-GCTGATGGCGATGAATGAACACTG-3′
feCath sense	5′-TTCAACCAGCGGTCCTCAGAGAAG-3′
feCath antisense	5′-TCACCAGCCCATTGTCCTTG-3′
feRPS5 sense	5′-ACAGTGCCCCATTGTGGAGC-3′
feRPS5 antisense	5′-AATAATCGCATTCACCAAGACCTG-3′

### PCR Amplification of feCath

PCR primers were designed using MacVector software (MacVector Inc, Cary, NC) to specifically amplify feCath (Table 1: feCath sense, feCath antisense) and the housekeeping transcript, feline ribosomal protein S5 (feRPS5) that was previously evaluated in dogs [21] (Table 1: feRPS5 sense, feRPS5 antisense). For both genes, sense and antisense primers were selected from different exons so that amplification of cDNA could be easily distinguished from amplification from genomic DNA. Qualitative PCR using FastStart Taq (Roche Applied Sciences, Indianapolis, IN) using the following parameters: 94°C for 5 min; 35 cycles of 94°C for 30 sec, 55°C for 30 sec, and 72°C for 1 min; and a final extension at 72°C for 7 min. PCR products were resolved by agarose gel electrophoresis (1.5% w/v) and detected by ethidium bromide staining. Quantitative RT-PCR was performed on cDNA representing 10 ng of total RNA per reaction using Lightcycler FastStart DNA MasterPLUS SYBR Green I (Roche) with the following parameters: 95°C for 5 min; 45 cycles of 95°C for 10 sec, 58°C for 5 sec, and 72°C for 12 sec; and a subsequent melting curve that ramped the temperature from 72°C to 95°C in increments of 0.1°C/sec. Non-template controls contained water, and gene-specific plasmid standards were used both as positive controls and for absolute quantification. All samples were performed in duplicate and variation between duplicates was <10% for every reported value.

### Immunocytochemistry

To lyse feline red blood cells, 7 ml of an ammonium chloride lysis solution (0.8% (w/v) NH_4_Cl/0.1 M EDTA in water, Stem Cell Technologies, Vancouver, BC, Canada) was added to 1 ml of peripheral blood and placed on ice for 15 min. The resulting solution was centrifuged at 410×g for 7 minutes to pellet leukocytes, and supernatant was discarded. Pelleted leukocytes were washed twice with 5 ml of phosphate buffered saline (PBS) and centrifuged at 410×g for 7 minutes with each wash. Cells were resuspended in 1 ml of fresh PBS, and 100 µl of this suspension was added to individual cytospin setups. The cytospins were centrifuged for 5 min and the apparatus was carefully removed, slides were allowed to dry and fixed in ethanol. Endogenous peroxide activity was eliminated by treating the slides with 0.1% hydrogen peroxide in methanol for 3 min. Slides were treated once with 95% ethanol for 2 min and once with 70% for 2 min, and blocked with 0.02% goat serum in PBS. Slides were incubate overnight at 4°C with 1∶500 dilution of mCRAMP antibody diluted in 0.02% goat serum in PBS [20]. Negative control slides were incubated with 0.02% goat serum in PBS overnight at 4°C. Following the overnight incubation, slides were washed with PBS twice for 10 min. Biotinylated α-rabbit secondary antibody (Vector Elite Kit, Vector Laboratories, Burlingame, CA) was applied for 20 min and subsequently washed with PBS twice for 2 min. ABC reagent was applied to the slides and incubated for 20 min at room temperature. Slides were washed with tris-buffered saline (TBS) three times for 2 min. Cytospins were developed using DAB solution for 5 min, washed with TBS for 4 min and water for 4 min, and lastly counterstained with Geimsa for 10 min. Counterslips were applied and images recorded using an Olympus BX-40 microscope.

### Peptide Synthesis, Purification, Determination of Concentration

feCath peptide (QLGELIQQGGQKIVEKIQKIGQRIRDFFSNLRPRQEA) was synthesized by CPC Scientific (San Jose, CA). The synthesized peptide was further purified with reverse phase high performance liquid chromatography (RP-HPLC) using a 0-80% gradient Buffer B (99.9% acetonitrile, 0.1% trifluoroacetic acid), measuring absorbance at 214 nm. The fraction eluting at minute 29 was collected and used in subsequent reactions as feCath. Synthetic LL-37 (LLGDFFRKSKEKIGKEFKRIVQRIKDFLRNLVPRTES) was used as a comparison for activity[22]. Approximately 1 ug of each peptide was added to 3X protein sample buffer (5% acetic acid, 3 M urea, 0.01% methyl green) and loaded into a 12.5% acid urea polyacrylamide gel electrophoresis (AU-PAGE) and run at 130 V for 1.5 hours. The resulting gel was stained with Simply Blue (Invitrogen) and destained with water.

#### Circular Dichroism

CD spectra were acquired using an Applied Photophysics Chirascan (Applied Photophysics, Surrey, UK) at 25°C at a peptide concentration of 50 µM in either water or 10 mM SDS. Data were obtained from 190–320 nm at 0.5 nm intervals with a bandwidth of 1 nm. Helical content was determined by visual inspection of the ellipticity at wavelengths of 208 nm and 222 nm [23].

### DNA Binding Assay

Hae-III digested phage DNA Phi-X174 (20 ng/µl) (Roche) was incubated with varying amounts of LL-37, feCath and magainin (10 ng/µl, 30 ng/µl and 100 ng/µl) for 5 min at room temperature. The entire reaction was resolved using agarose gel electrophoresis (1% w/v) and stained with ethidium bromide.

### Antimicrobial Assay

A radial diffusion antimicrobial assay was used to assess the antimicrobial activity of feCath and LL-37 [24]. Briefly, overnight bacterial cultures were seeded into fresh tryptic soy broth (TSB). Bacteria was grown to an optical density of 0.2 at A_600_, pelleted and resuspended in an equal volume of 10 mM sodium phosphate buffer, pH 7.4. Resuspended bacteria were added to underlay minimal-media agarose, and peptide, at various concentrations (1000 ng/µl, 333 ng/µl, 111 ng/µl, 37 ng/µl, 12 ng/µl and 4 ng/µl, dissolved in 0.05% acetic acid), was instilled into adjacent punch-wells. Buffer alone (0.05% acetic acid) was used as the negative control. After 3 hours of incubation at 37°C, overlay nutrient rich agarose was added and allowed to incubate overnight. The following day, zones of clearance were measured. To quantitate zones of clearance, the diameter of the agarose punch (3 mm) was subtracted from the diameter of antimicrobial clearance (in mm), and the resulting number was multiplied by 10.

## Results

### Identification of feCath cDNA and gene sequence

**Figure 1:**
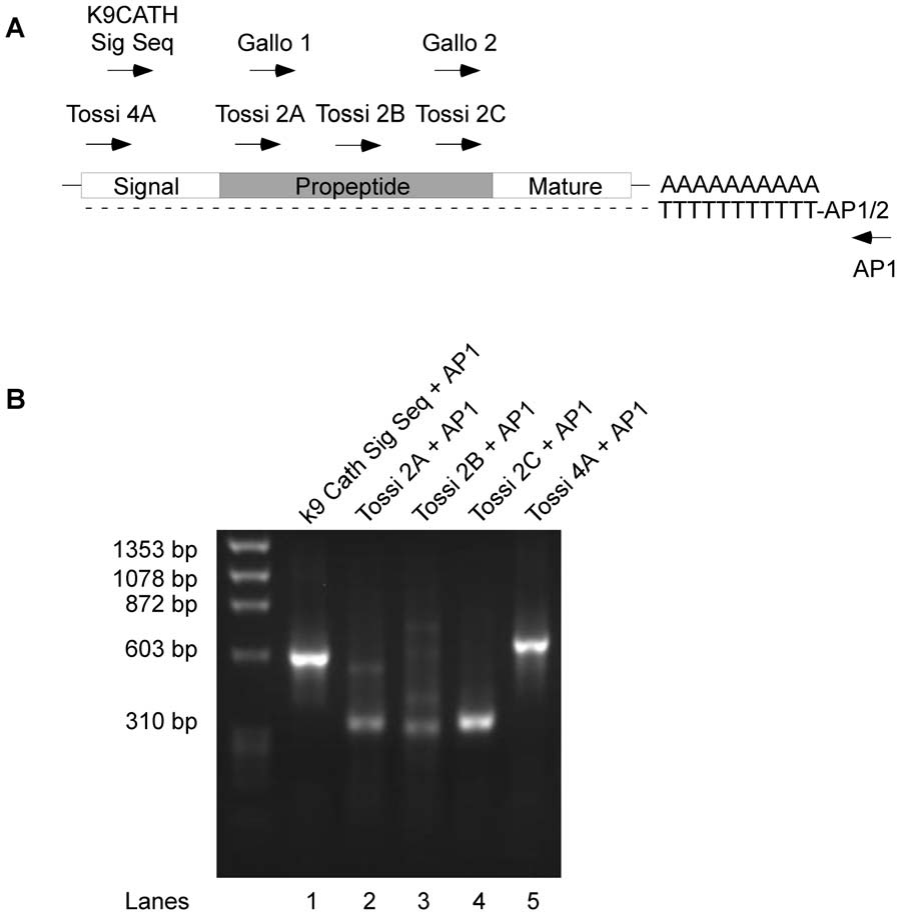
3′ RACE Analysis and Identification of feCath. (A) Schematic of 3′ RACE strategy targeting the signal sequence region and the propeptide (cathelin) domain with sense primers and using antisense adaptor primer, AP1. Feline bone marrow RNA was reverse transcribed with oligo-dT conjugated to adaptor primer 1/2 (AP1/2). Sense primers (sequences found in Table 1) were used to amplify cathelicidin related sequences in the pool of bone marrow cDNA. (B) Agarose gel electrophoresis analysis of 3′ RACE PCR products from (A). HaeIII digested Phi-X174 phage DNA used as the marker.

**Figure 2:**
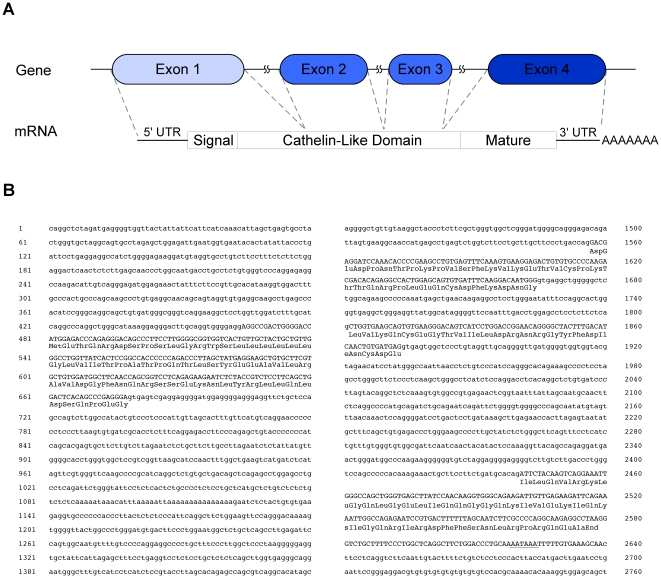
Nucleotide sequence of feCath gene and flanking region. (A) Gene and mRNA schematic of feCath showing four exons and interspersed intronic sequences. The mRNA sequence includes for the 5′ and 3′ untranslated regions, a coding region for signal sequence, cathelin-like domain, mature peptide, and a polyadenylated tail. (B) Nucleotide sequence of *feCath* gene and flanking regions. Numbering begins arbitrarily at the 5′ most nucleotide. Lowercase letters indicate intronic and flanking sequence, uppercase letters indicate exonic sequence with deduced amino acid sequence of coding region below in three-letter code. Polyadenylation signal is underlined. The cDNA sequence was deposited in GenBank (Accession #: HQ221766).

To identify cathelicidins expressed in cats, a 3′-RACE strategy was employed using sense PCR primers that anneal to sequences encoding either the signal sequence or the propeptide domains (Figure 1A) [19], [20]. These regions are typically highly conserved among cathelicidins. For these experiments, either a primer based on the signal sequence region of the canine cathelicidin, K9CATH, or primers targeting for the cathelin-like domain from other species were used (Figure 1B) [19]. PCR products were generated from bone marrow cDNA using these sense primers and an antisense primer to the 3′-anchor sequence as described in methods. The PCR products were either subcloned into pBluescript prior to sequencing (lane 1, 12 clones) or directly sequenced after silica gel adsorption chromatography (lanes 2–5). All sequences were aligned and only one single sequence was identified irrespective of the 3′-RACE strategy employed. This supports that the cat encodes a single feline cathelicidin, we named feCath. Polymorphisms were detected in the signal sequence and propeptide, however, none were detected in the mature peptide sequence (data not shown). A 5′-RACE strategy using a feCath specific antisense primer and adaptor primers provided sequence data extending to the 5′ end of the transcript. *In silico* analysis of the composite feCath mRNA sequence, using the UCSC BLAST-like Alignment Tool (BLAT), revealed that the feCath gene consists of four exons, a consistent feature with all other cathelicidins identified (Figure 2A and 2B). Using sequences from each exon, UCSC BLAT searches revealed only one gene match with the feline genome, furthering the proposed existence of only a single cathelicidin in cats. NCBI BLAST searches of sequence from each exon yielded no feline sequences, likely due to incomplete information on the cat genome in this database.

The feCath mRNA contains a single open reading frame of 593 nucleotides. The deduced amino acid sequence predicts a 171 amino acid prepropeptide consisting of a putative signal sequence region, a cathelin-like domain and a mature peptide (Figure 2A). The mature peptide has a predicted length of 37 amino acids based on comparisons with other closely related cathelicidins of dogs, mice, rats and humans.

### Sequence Comparison

**Figure 3:**
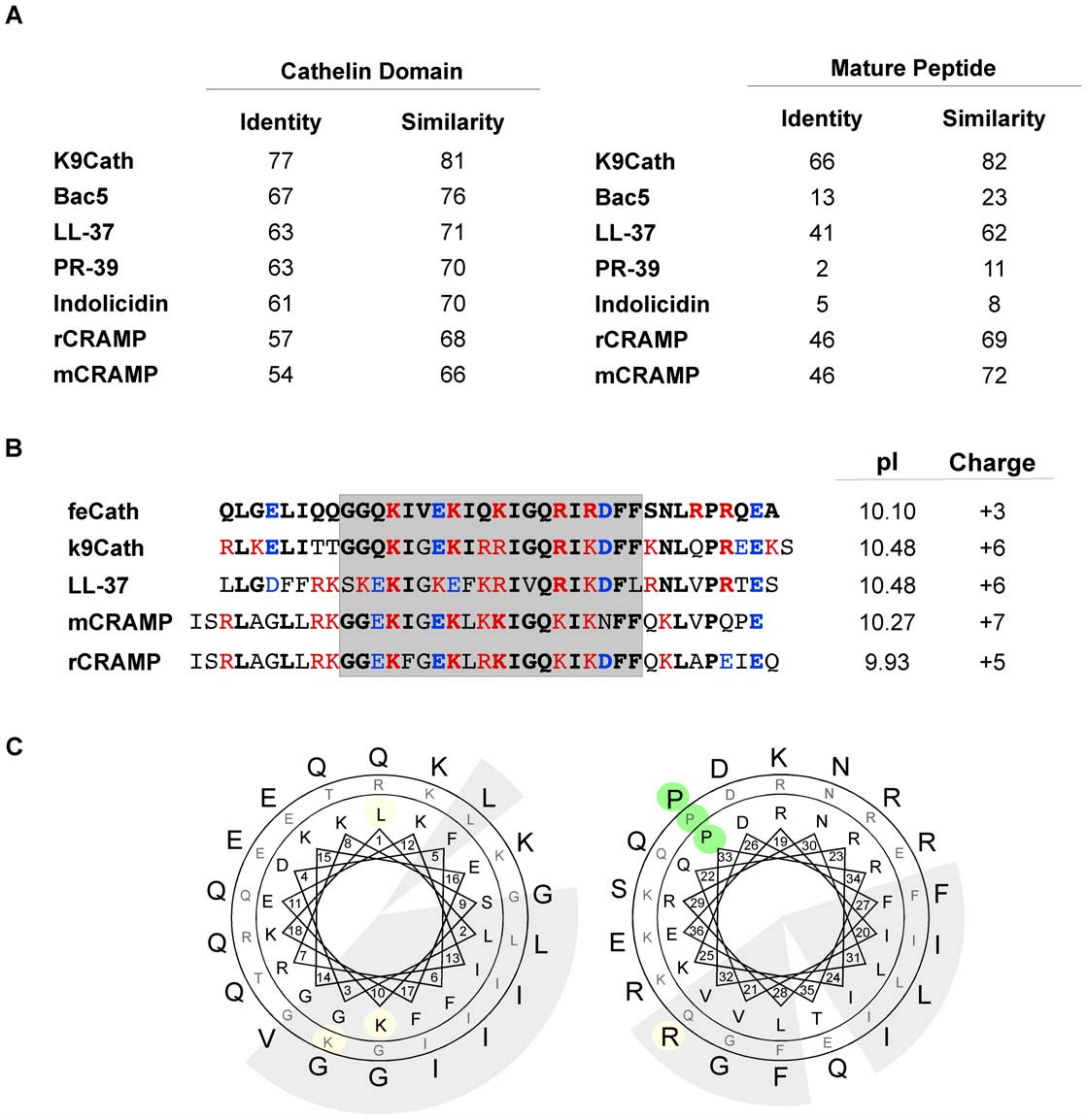
Amino acid comparison of cathelin-like domain and mature cathelicidin. (A) Table of sequence identity and similarity of the cathelin-like domain from diverse groups of cathelicidins and the mature peptide of more closely related cathelicidins (PR-39 for distant comparison with mature peptides). K9CATH: dog, Bac5: cow, LL-37: human, PR-39: pig, Indolicidin: cow, mCRAMP: mouse, rCRAMP: rat. (B) Amino acid alignment of closely related mature cathelicidins. Residues in red are basic, residues in blue are acidic, and bolded residues are identical feCath sequence. pI and net charge are calculated for each peptide. Grey box indicates region of high similarity, and maintenance of cationic and hydrophobic residues. (C) Hydrophobic and polar residue clusters shown on a helical wheel projection. Based on the same analytical approach of Zelezetsky *et al* [25], sequence of feCath (outer ring) is compared to K9CATH (middle ring) and human LL-37 (inner ring). The analysis is divided into N- and C-terminal sequences, with residues 1–18 shown in the left wheel and 19–36 in the right wheel. Clusters of nonpolar residues are on a shaded background. Residues that deviate from the pattern of the three sequences are highlighted. The helical-breaking proline residue near the C-terminus is on a green background.

Cathelicidins are a unique family of peptides in that they contain a highly conserved propeptide domain, whereas the sequence of the mature peptide can be widely divergent. Indeed, the cathelin-like propeptide domain of feCath is highly similar to the corresponding domain from diverse cathelicidin subgroups: mice (mCRAMP, 66%), rats (rCRAMP, 68%), pigs (PR-39, 70%), cows (Indolicidin, 70% and Bac5, 76%), humans (LL-37, 71%), and dogs (K9CATH, 81%). However, the mature peptide has similarity in amino acid sequence to only a subset of cathelicidins including: dogs (K9CATH, 82%), humans (LL-37, 62%), mice (mCRAMP, 72%) and rats (rCRAMP, 69%) (Figure 3A). In contrast, there is little similarity with cathelicidins from other subgroups: cows (Bac5, 23% and Indolicidin, 8%) and pigs (PR-39, 11%). Further analysis of the mature peptide sequence from cats, dogs, humans, mice and rats, revealed a highly similar core sequence with conserved cationic and hydrophobic residues (Figure 3B, grey box). Despite a lower overall net charge of +3 when compared to other cathelicidins in this subgroup, feCath has a relatively high pI of 10.10, owing to preponderance of arginine residues (Figure 3B). Based on these considerations, feCath can be classified as a cathelicidin mostly resembling the linear subgroup.

Tossi and colleagues have analyzed the amphipathic characteristics of LL-37 and other members of the linear α-helix subgroup of cathelicidins. In an analysis of 20 non-human primate sequences, residue variations tended to maintain well-demarcated polar and hydrophobic surfaces along the α-helical axis, even though charge distribution varied [25]. Using the same helical wheel projection, feCath maintains this pattern (Figure 3C). Based on this comparison with two peptides that experimentally are known to form a helical secondary structure [7], [26], we predicted that feCath similarly forms an amphipathic α-helix, which was confirmed as described below.

### Tissue expression

**Figure 4:**
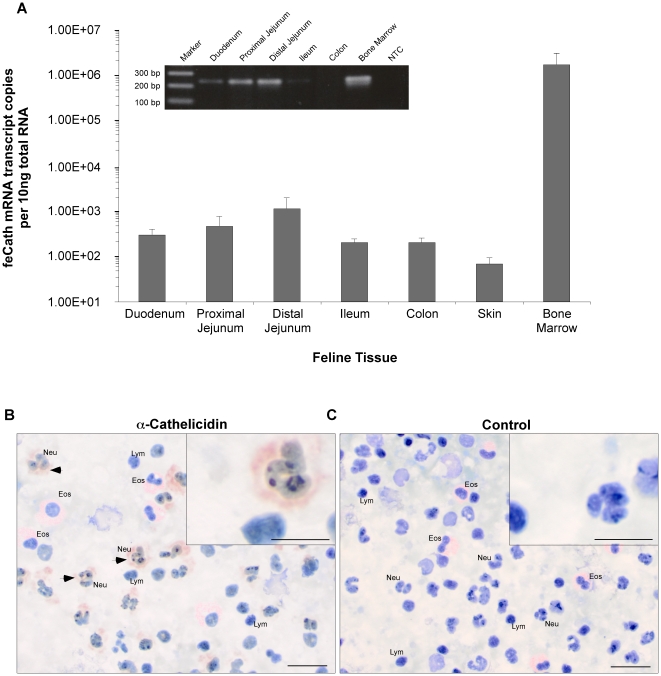
Expression and immunolocalization of feline cathelicidin. (A) Quantitative RT-PCR (qPCR) analysis of absolute copy number of mRNA transcripts encoding feCath in RNA isolated from duodenum (n = 8), proximal jejunum (n = 7), distal jejunum (n = 8), ileum (n = 8), colon (n = 8), skin (n = 2) and bone marrow (n = 7). Non-template control (NTC) in qPCR yielded <101 copies per 10 ng. Error bars represent standard error. Inset is a representative gel electrophoresis of feCath amplification in gastrointestinal tract and bone marrow. Expected size based on primer location is 234 bp. (B) Immunostaining for feCath on feline leukocytes using a polyclonal antibody (1∶500) raised against mCRAMP. feCath localizes to neutrophils in the peripheral circulation. (C) Negative control for staining with secondary antibody only. Images taken at 50X (bar: 20 µm), inset taken at 100X (bar: 10 µm). **Key**, Eos: eosinophils, Neu: neutrophils, Lym: lymphocytes, arrow: feCath staining.

To determine the distribution of tissue expression, total RNA was isolated from gastrointestinal tract (duodenum, jejunum, ileum and colon), skin and bone marrow, and reverse transcribed into cDNA. PCR primer pairs were designed to specifically amplify cDNA of feCath. Other primers specifically amplified cDNA of the housekeeping gene, feRPS5 [21]. High levels of feCath expression was detected in the bone marrow, with levels greater than 1.00E6 transcript copies per 10 ng total RNA, whereas lower expression was detected in gastrointestinal tissues and skin (Figure 4A). feRPS5 mRNA expression was consistently detected at similar levels between tissues and individual cats (data not shown). Expression of feCath peptide was analyzed in peripheral leukocytes using a polyclonal antibody raised against the mouse cathelicidin, mCRAMP. Immunostaining was detected in the cytoplasm of neutrophils (Figure 4B), but not in other leukocytes. No immunostaining was observed in the cytoplasm of neutrophils in control experiments lacking primary antibody (Figure 4C). Thus, the expression pattern of feCath is consistent with that for other previously identified cathelicidins, where mRNA expression is high in the bone marrow and peptide is observed in neutrophils [8], [20], [26], [27].

### Peptide Structure and Antimicrobial Activity

**Figure 5:**
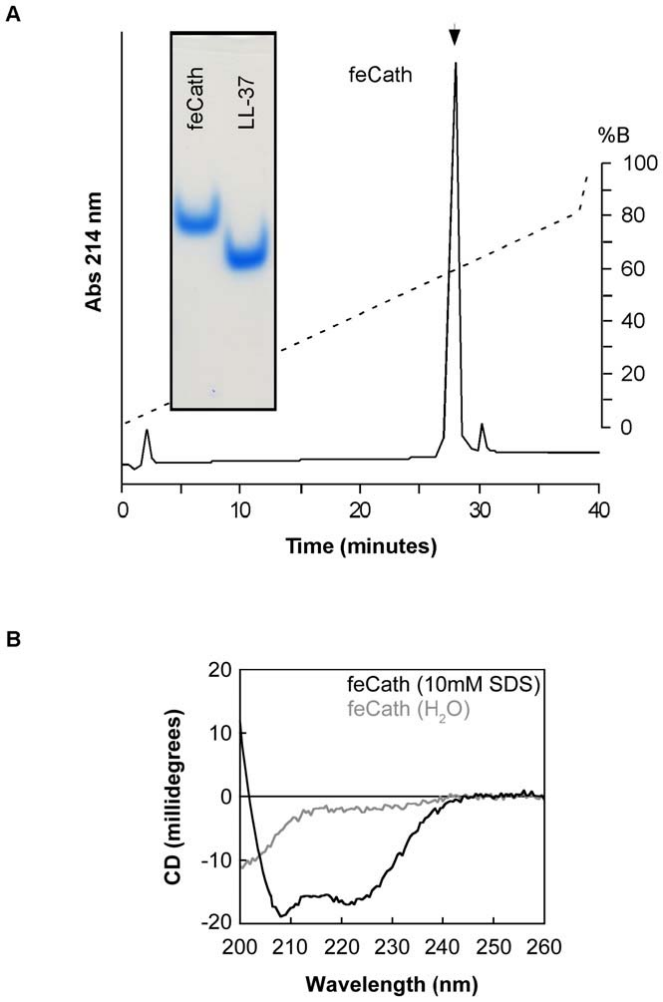
Purification and Structure of feCath. (A) Reverse phase HPLC purification of synthetic feCath using a gradient of acetonitrile. Dotted line and inset indicate elution gradient. Arrow indicates positive fraction for feCath. AU-PAGE of synthetic LL-37 and feCath showing peptide homogeneity and equal peptide concentrations by Simply Blue staining (inset). (B) CD spectra of feCath (50 µM) in water (gray line) and 10 mM SDS (black line).

The predicted mature feCath was chemically synthesized and purified with reverse-phase high performance liquid chromatography (RP-HPLC) (Figure 5A). The peptide had a mass of 4323.5 Da (expected: 4322.7 Da). The purified feCath preparation was compared to synthetic LL-37 by acid urea polyacrylamide gel electrophoresis (AU-PAGE) to assess homogeneity and confirm peptide concentration (Figure 5A, inset). Using circular dichroism, the peptide secondary structure was evaluated in both water and 10 mM SDS. feCath adopted a random structure in water; however, when SDS was added feCath assumed an α-helical secondary structure, evident by the negative ellipicity at 208 nm and 222 nm (Figure 5B). The properties of feCath in both a water and lipophilic environment are similar to that of LL-37 under these conditions [25]. Antimicrobial activity of this feCath preparation was determined using a radial diffusion antimicrobial assay on both Gram-positive and Gram-negative bacteria. Human LL-37 was used as a reference to compare antimicrobial activity. feCath exhibited antimicrobial activity against Gram-positive bacteria: *Listeria monocytogenes* and a clinical isolate of *Staphylococcus pseudintermedius* (MIC: 11 and 12 µg/ml, respectively) and Gram-negative bacteria: *E. coli* D31 and *Salmonella enterica* serovar Typhimurium (MIC: 21 and 29 µg/ml) bacteria (Table 2). This activity was similar to LL-37 (MIC: 6-15 µg/ml), but not as potent against some of the bacteria, such as *Staphylococcus pseudintermedius*.

**Table 2:** Antibacterial acivity.

	MIC (µg/ml)	
Bacteria	LL-37	feCath
*E. coli* D31	5	21
*Salmonella enterica* serovar Typhimurium (IR715)	12	29
*Listeria monocytogenes*	7	12
*Staphylococcus pseudointermedius* (clinical isolate)	15	11

Minimum inhibitory concentration (MIC) of feCath and LL-37 using a radial diffusion antimicrobial assay.

### DNA Binding Activity

**Figure 6:**
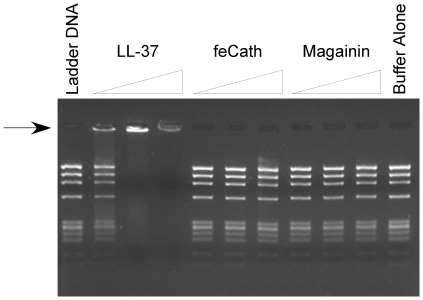
Gel electrophoresis of DNA binding assay. A DNA ladder (HaeIII digested Phi-X174, 200 ng) was incubated with increasing concentrations of peptide (100 ng, 300 ng, 1000 ng) for 5 minutes in 0.05% acetic acid at room temperature. The reaction mixture was resolved using agarose gel electrophoresis (2% w/v). Arrow indicates retarded migration of Phi-X174 DNA into the gel.

LL-37 binds to host DNA [16]. This DNA-peptide complex can exacerbate inflammation when detected by plasmacytoid dendritic cells, triggering TLR9 signaling and inducing release of type I interferon. To test whether this is a general activity of linear cathelicidins, feCath was incubated with HaeIII digested Phi-X174 phage DNA, and gel electrophoresis was performed. LL-37 was able to bind the DNA and retard its migration into the gel in a dose dependent manner. Interestingly, feCath, despite its similar pI, was not able to retard the migration of DNA under identical conditions, irrespective of the amount of peptide (Figure 6). Magainin, another linear, cationic antimicrobial peptide (pI: 9.98) found in the skin of frogs, also lacks this ability to bind DNA. Therefore, these results suggest that feCath does bind DNA despite its cationic properties, as well as its sequence and structural similarities to human LL-37.

## Discussion

Cathelicidins are a diverse group of antimicrobial peptides characterized from many species including pigs [28], cows [29], sheep [30], mice [20], rats [31], horses [32], rabbits [33], dogs [26], non-human primates [25] and humans [7]. While the mature cathelicidins are extremely diverse in sequence, the unifying feature of this peptide family is conserved the propeptide, the cathelin-like domain [3]. Another curious feature of cathelicidins is that some species have a single-family member (such as dogs, mice, rats and humans), while other species have five or more distinct representatives [6]. This study sought to identify and characterize cathelicidin(s) encoded by the feline genome. Using a combination of strategies, we identified a single cathelicidin, named feCath, and detected its expression to extremely high levels in the bone marrow and localized the peptide to neutrophils.

In species whose genome encodes only a single cathelicidin, the mature peptide is a linear α-helix [6]. Consistent with this pattern, feCath is a linear peptide with high sequence similarity to this subgroup of cathelicidins. The distribution of polar and nonpolar hydrophobic residues predicts that, like LL-37 [25] and other members of this subgroup, feCath has an amphipathic secondary structure. In the presence of a lipophilic environment (SDS), feCath adopts an α-helix, similar to LL-37 [25]. The mature peptides of this subgroup, including feCath, have potent antimicrobial activity against Gram-positive and Gram-negative bacteria [6], [34]. We should note that the N-terminus of cathelicidins can affect their antimicrobial activity and the current study did not address this possible variation for feCath, but rather assessed activity of peptide corresponding to the major human isoform. In addition to this similarity in mature peptide sequence and activity, all members of this subfamily of cathelicidins are expressed in neutrophils. Thus, feCath can be categorized with other cathelicidins of the α-linear helix group that includes LL-37, K9CATH, mCRAMP and rCRAMP.

The regulation of human hCAP18/LL-37 expression is cell-type specific. For example, hCAP18/LL-37 expression in the bone marrow is constitutively high. In contrast, vitamin D_3_ can induce the expression of hCAP18/LL-37 mRNA in human keratinocytes and monocytes [13]. Analysis of the promoter of the hCAP18/LL-37 gene has revealed the presence of a vitamin D response element (VDRE) approximately 700 bp upstream of the transcription start site [35]. The VDRE is also conserved in short interspersed nuclear elements (SINE) found in the *CAMP* promoter of non-human primates; however, a comparative analysis with mice, rats and canine genomes indicated that this SINE element is not present in any of these species [35]. Gombart *et al* substantiated this genomic sequence analysis with experimental data, by showing that treatment of mouse bone marrow cells with vitamin D_3_ did not increase mCRAMP levels [35]. Additionally, vitamin D receptor knockout mice reduced mCRAMP expression, when compared with wildtype mice [35]. In the 5′ flanking region of the *feCath* genomic sequence, we were unable to find a VDRE consensus sequence, suggesting that feCath is not inducible with vitamin D_3_ treatment. Therefore, exogenous treatment of feline skin with vitamin D_3_, to increase the feCath expression, may not be clinically effective.

Human LL-37 has the ability to bind to host DNA and serve as an activating ligand for TLR9 in plasmacytoid dendritic cells [16]. The subsequent signaling cascade results in the upregulation of type I IFN [16]. In chronic inflammatory disorders, such as psoriasis, this activity is thought to exacerbate the existing inflammation [16]. From our study, feCath lacks a comparable ability to bind DNA. If the results of our *in vitro* study recapitulated *in vivo* properties of this peptide, feCath in the cat would not play a role similar to LL-37 in the exacerbation of inflammation through DNA binding activity. Other pathways by which feCath might affect type I IFN (if any) were not investigated in this study.

Overall, we report a novel cathelicidin of the domestic cat, which we have named feCath. Due to the similarities in sequence, expression patterns and antimicrobial activity, feCath belongs to the linear α-helix subgroup of cathelicidins found in dogs, mice, rats and humans. feCath has similar antimicrobial activity to LL-37, yet lacks the ability to bind DNA. Given these attributes, studies on the structure and function of the mature peptide will advance our knowledge of the critical sequences and domains necessary for activity.

## References

[1] Zasloff M (2002). Antimicrobial peptides of multicellular organisms.. Nature.

[2] Lehrer RI (2004). Primate defensins.. Nat Rev Microbiol.

[3] Zanetti M, Gennaro R, Romeo D (1995). Cathelicidins: a novel protein family with a common proregion and a variable C-terminal antimicrobial domain.. FEBS Lett.

[4] Zaiou M, Nizet V, Gallo RL (2003). Antimicrobial and protease inhibitory functions of the human cathelicidin (hCAP18/LL-37) prosequence.. J Invest Dermatol.

[5] Verbanac D, Zanetti M, Romeo D (1993). Chemotactic and protease-inhibiting activities of antibiotic peptide precursors.. FEBS Lett.

[6] Zanetti M (2005). The role of cathelicidins in the innate host defenses of mammals.. Curr Issues Mol Biol.

[7] Agerberth B, Gunne H, Odeberg J, Kogner P, Boman HG (1995). FALL-39, a putative human peptide antibiotic, is cysteine-free and expressed in bone marrow and testis.. Proc Natl Acad Sci U S A.

[8] Cowland JB, Johnsen AH, Borregaard N (1995). hCAP-18, a cathelin/pro-bactenecin-like protein of human neutrophil specific granules.. FEBS Lett.

[9] Agerberth B, Charo J, Werr J, Olsson B, Idali F (2000). The human antimicrobial and chemotactic peptides LL-37 and alpha-defensins are expressed by specific lymphocyte and monocyte populations.. Blood.

[10] Frohm M, Agerberth B, Ahangari G, Stahle-Backdahl M, Liden S (1997). The expression of the gene coding for the antibacterial peptide LL-37 is induced in human keratinocytes during inflammatory disorders.. J Biol Chem.

[11] Frohm Nilsson M, Sandstedt B, Sorensen O, Weber G, Borregaard N (1999). The human cationic antimicrobial protein (hCAP18), a peptide antibiotic, is widely expressed in human squamous epithelia and colocalizes with interleukin-6.. Infect Immun.

[12] Turner J, Cho Y, Dinh NN, Waring AJ, Lehrer RI (1998). Activities of LL-37, a cathelin-associated antimicrobial peptide of human neutrophils.. Antimicrob Agents Chemother.

[13] Schauber J, Dorschner RA, Yamasaki K, Brouha B, Gallo RL (2006). Control of the innate epithelial antimicrobial response is cell-type specific and dependent on relevant microenvironmental stimuli.. Immunology.

[14] Liu PT, Stenger S, Li H, Wenzel L, Tan BH (2006). Toll-like receptor triggering of a vitamin D-mediated human antimicrobial response.. Science.

[15] De Y, Chen Q, Schmidt AP, Anderson GM, Wang JM (2000). LL-37, the neutrophil granule- and epithelial cell-derived cathelicidin, utilizes formyl peptide receptor-like 1 (FPRL1) as a receptor to chemoattract human peripheral blood neutrophils, monocytes, and T cells.. J Exp Med.

[16] Lande R, Gregorio J, Facchinetti V, Chatterjee B, Wang YH (2007). Plasmacytoid dendritic cells sense self-DNA coupled with antimicrobial peptide.. Nature.

[17] Ong PY, Ohtake T, Brandt C, Strickland I, Boguniewicz M (2002). Endogenous antimicrobial peptides and skin infections in atopic dermatitis.. N Engl J Med.

[18] Wehkamp J, Chu H, Shen B, Feathers RW, Kays RJ (2006). Paneth cell antimicrobial peptides: topographical distribution and quantification in human gastrointestinal tissues.. FEBS Lett.

[19] Tossi A, Scocchi M, Zanetti M, Gennaro R, Storici P, Romeo D, Shafer WM (1997). Antimicrobial Peptide Protocols;.

[20] Gallo RL, Kim KJ, Bernfield M, Kozak CA, Zanetti M (1997). Identification of CRAMP, a cathelin-related antimicrobial peptide expressed in the embryonic and adult mouse.. J Biol Chem.

[21] Brinkhof B, Spee B, Rothuizen J, Penning LC (2006). Development and evaluation of canine reference genes for accurate quantification of gene expression.. Anal Biochem.

[22] Yamasaki K, Di Nardo A, Bardan A, Murakami M, Ohtake T (2007). Increased serine protease activity and cathelicidin promotes skin inflammation in rosacea.. Nat Med.

[23] Greenfield NJ (2006). Using circular dichroism spectra to estimate protein secondary structure.. Nat Protoc.

[24] Lehrer RI, Rosenman M, Harwig SS, Jackson R, Eisenhauer P (1991). Ultrasensitive assays for endogenous antimicrobial polypeptides.. J Immunol Methods.

[25] Zelezetsky I, Pontillo A, Puzzi L, Antcheva N, Segat L (2006). Evolution of the primate cathelicidin. Correlation between structural variations and antimicrobial activity.. J Biol Chem.

[26] Sang Y, Teresa Ortega M, Rune K, Xiau W, Zhang G (2007). Canine cathelicidin (K9CATH): gene cloning, expression, and biochemical activity of a novel pro-myeloid antimicrobial peptide.. Dev Comp Immunol.

[27] Zanetti M, Del Sal G, Storici P, Schneider C, Romeo D (1993). The cDNA of the neutrophil antibiotic Bac5 predicts a pro-sequence homologous to a cysteine proteinase inhibitor that is common to other neutrophil antibiotics.. J Biol Chem.

[28] Agerberth B, Lee JY, Bergman T, Carlquist M, Boman HG (1991). Amino acid sequence of PR-39. Isolation from pig intestine of a new member of the family of proline-arginine-rich antibacterial peptides.. Eur J Biochem.

[29] Romeo D, Skerlavaj B, Bolognesi M, Gennaro R (1988). Structure and bactericidal activity of an antibiotic dodecapeptide purified from bovine neutrophils.. J Biol Chem.

[30] Huttner KM, Lambeth MR, Burkin HR, Burkin DJ, Broad TE (1998). Localization and genomic organization of sheep antimicrobial peptide genes.. Gene.

[31] Termen S, Tollin M, Olsson B, Svenberg T, Agerberth B (2003). Phylogeny, processing and expression of the rat cathelicidin rCRAMP: a model for innate antimicrobial peptides.. Cell Mol Life Sci.

[32] Scocchi M, Bontempo D, Boscolo S, Tomasinsig L, Giulotto E (1999). Novel cathelicidins in horse leukocytes(1).. FEBS Lett.

[33] Ooi CE, Weiss J, Levy O, Elsbach P (1990). Isolation of two isoforms of a novel 15-kDa protein from rabbit polymorphonuclear leukocytes that modulate the antibacterial actions of other leukocyte proteins.. J Biol Chem.

[34] Gennaro R, Zanetti M (2000). Structural features and biological activities of the cathelicidin-derived antimicrobial peptides.. Biopolymers.

[35] Gombart AF, Borregaard N, Koeffler HP (2005). Human cathelicidin antimicrobial peptide (CAMP) gene is a direct target of the vitamin D receptor and is strongly up-regulated in myeloid cells by 1,25-dihydroxyvitamin D3.. FASEB J.

